# Construction of a high-density genetic map for yardlong bean and identification of ANT1 as a regulator of anthocyanin biosynthesis

**DOI:** 10.1093/hr/uhad247

**Published:** 2023-11-27

**Authors:** Hongmei Zhang, Wei Zhang, Shan Meng, Linchong Hui, Xiaoqing Liu, Wei Chen, Wei Yan, Xin Chen, Huatao Chen

**Affiliations:** Institute of Industrial Crops, Jiangsu Academy of Agricultural Sciences/Jiangsu Key Laboratory for Horticultural Crop Genetic Improvement, Nanjing 210014, China; Institute of Industrial Crops, Jiangsu Academy of Agricultural Sciences/Jiangsu Key Laboratory for Horticultural Crop Genetic Improvement, Nanjing 210014, China; Institute of Germplasm Resources and Biotechnology, Jiangsu Academy of Agricultural Sciences, Nanjing 210014, China; Lianyungang Institute of Agricultural Sciences, Jiangsu Academy of Agricultural Sciences, Lianyungang, 222000, China; Institute of Industrial Crops, Jiangsu Academy of Agricultural Sciences/Jiangsu Key Laboratory for Horticultural Crop Genetic Improvement, Nanjing 210014, China; Lianyungang Institute of Agricultural Sciences, Jiangsu Academy of Agricultural Sciences, Lianyungang, 222000, China; Institute of Germplasm Resources and Biotechnology, Jiangsu Academy of Agricultural Sciences, Nanjing 210014, China; Institute of Industrial Crops, Jiangsu Academy of Agricultural Sciences/Jiangsu Key Laboratory for Horticultural Crop Genetic Improvement, Nanjing 210014, China; Institute of Industrial Crops, Jiangsu Academy of Agricultural Sciences/Jiangsu Key Laboratory for Horticultural Crop Genetic Improvement, Nanjing 210014, China

## Abstract

Because its long, tender pods supply essential proteins, vitamins, and fibers to humans, yardlong bean (*Vigna unguiculata ssp. sesquipedalis*) is a commonly consumed vegetable, especially in Southeast Asia. To provide insights into the genetic bases of key agricultural traits in yardlong bean, we here created a high-density bin-map with 2084 bin markers using 514 227 SNPs from a recombinant-inbred line (RIL) population. Quantitative trait loci (QTL) mapping was carried out to identify loci associated with anthocyanin content (ANT), vitamin E content (VE), total soluble protein content (TSP), pod length (PL), hundred-seed weight (HSW), seed length and width (SL and SW, respectively), and seed coat color (SCC). In total, 20 related QTLs were isolated, explaining 7.58–56.03% of the phenotypic variation. Of these, five major QTLs (*qANT5*, *qTSP11*, *qVE7*, *qPL3*, and *qSCC9*) were detected in 2020, 2021, and the combined environment, explaining 11.96–56.03% of the phenotypic variation. *VuANT1* was identified as a causal gene for the QTL *qANT5*, which regulated anthocyanin content; *VuANT1* was highly expressed in immature purple pods but barely detectable in white pods. *VuANT1* overexpression in tobacco leaves and yardlong bean hairy roots resulted in purple coloration as a result of anthocyanin accumulation. These findings suggested that *VuANT1* was a key regulator of anthocyanin accumulation in yardlong bean. Our results lay a firm foundation for target agricultural trait improvement and clarification of the genetic mechanisms underlying agricultural traits in yardlong bean.

## Introduction

As a diploid warm-season legume, *Vigna unguiculata* L. Walp is economically important in Africa, America, and Asia [1, 2]. There are two major cultivar groups: *V. unguiculata ssp. unguiculata* (commonly known as cowpea or black-eyed pea) and *V. unguiculata ssp. sesquipedalis* (also called asparagus bean, snake bean, or yardlong bean). Cowpea is most commonly grown in Africa, where typically only the dry seeds are collected for cooking and consumption, although occasionally the young pods or leaves are harvested [3]. Yardlong bean is principally grown in Asia, where both the pods and seeds are regularly consumed in fresh and cooked forms.

*V. unguiculata* must be planted annually but can be harvested a total of two to four times per season, beginning ~50 d after sowing [4]. It serves as a good source of dietary protein, fiber, vitamins, and minerals [5, 6]. Furthermore, some cultivars have purple pods, which are high in anthocyanin content; anthocyanins have superior antioxidant capacities, which can protect the human body against cardiovascular disease, aging-related illness, and some tumors [7–9]. Overall, the nutritional content, health benefits, capacity for multiple harvest, and ability to grow in sandy soil with low moisture make yardlong bean a highly valued crop.

Previous studies have sought to identify the genetic factors underlying specific characteristics of horticultural plant, including yardlong bean to enable optimization of key agronomically important traits [4, 10]. Such studies have yielded numerous quantitative trait loci (QTLs) for traits associated with organ size, yield potential, and flowering time. These studies have also revealed strong correlations between traits and co-localization of many key loci. For example, most of the QTLs responsible for seed, pod, stem, and leaf size were identified from LG7 [4]. QTL co-localization has also observed between pod tenderness and pod length and between total soluble solid content in the pods and pod dehiscence and/or length [11]. Yardlong bean quality is greatly influenced by the fiber bundle content of the pods, which appears to be primarily controlled by one major and one minor QTL, both of which are in the same physical region. Additional studies in yardlong bean and other legumes have identified QTLs associated with protein and vitamin E content, both of which are known to affect bean quality.

As mentioned above, some varieties of yardlong bean have purple pods that are high in anthocyanin content. Anthocyanins are a group of flavonoid pigments regulating the color of some flowers, fruits, seeds, and vegetables [12, 13]. Pod color was previously analysed in various legume species and was found to be linked to QTLs for the anthocyanin-biosynthesis pathway [14–16]. For example, using a set of 301 common bean lines from the Spanish Diversity Panel (SDP), one lab detected 18 QTLs for pod color, five of which were linked to eight candidate pigment synthesis genes [16]. In kidney bean, malvidin 3, 5-diglucoside 5-diglucoside was the main anthocyanin compound in the pod skin; expression levels of anythocyanin regulatory genes (such as *PvMYB1*, *PvMYB2*, and *PvTT8–1*) and most anthocyanin structural genes tend to be higher in the skin of purple pods than in pods of other colors [14]. In yardlong bean, expression profiles suggest that the genes *Vigun05g039400* and *Vigun05g039500* are responsible for seed coat color and that *Vigun05g039300* regulates pod tip color [17]. However, there have been no previous reports of QTLs for pod anthocyanin content in yardlong bean.

In the present study, we assessed the agronomic traits and nutritional quality of yardlong bean by measuring pod length, hundred-seed weight, seed size, seed coat color, and levels of anthocyanins, vitamin E, and total soluble proteins in a recombinant inbred line (RIL) population. After establishing a high-density genetic map for *V. unguiculata*, 20 QTLs were identified as associated with these key traits via linkage mapping. Moreover, experimental results suggested that *VnANT1* was the causal gene for the important anthocyanin-content QTL *qANT5*; it controlled anthocyanin accumulation by regulating genes that participate the anthocyanin biosynthetic pathway. These findings enhance our understanding of the genetic mechanisms responsible for yardlong bean nutritional quality and lay the foundation for future improvement of this economically important crop species.

**Table 1:** Bin marker located on the 11 chromosomes in the RIL population

Chr.	Chr. length	No. bin	Physical length (Mb)			Gap length (Mb)			Genetics distance (cM)		
	(Mb)		Sum	Average	Range	Sum	Average	Range	Sum	Average	Range
1	42.13	244	32.88	0.13	1 (bp)–2.50	9.25	0.04	19 (bp)–1.09	185.36	0.76	0.00–7.23
2	33.91	95	24.40	0.26	1 (bp)–3.46	9.51	0.10	10 (bp)–2.63	94.88	1.00	0.00–20.24
3	65.29	319	48.66	0.15	1 (bp)–4.12	16.64	0.05	2 (bp)–0.85	213.07	0.67	0.00–7.08
4	42.73	165	26.37	0.16	1 (bp)–2.52	16.36	0.10	5 (bp)–4.65	140.55	0.85	0.00–10.19
5	48.75	262	33.43	0.13	1 (bp)–1.43	15.32	0.06	8 (bp)–2.15	202.16	0.77	0.00–6.28
6	34.46	53	31.55	0.60	1 (bp)–8.81	2.91	0.06	20 (bp)–1.12	39.98	0.75	0.00–7.04
7	40.88	222	24.01	0.11	1 (bp)–1.49	16.86	0.08	7 (bp)–3.40	126.83	0.57	0.00–6.71
8	38.36	156	25.00	0.16	1 (bp)–3.10	13.36	0.09	3 (bp)–2.32	103.41	0.66	0.00–8.71
9	43.93	244	32.67	0.13	1 (bp)–4.79	11.26	0.05	3 (bp)–0.76	167.11	0.68	0.00–6.25
10	41.33	136	39.48	0.29	1 (bp)–3.47	1.84	0.01	2 (bp)–0.23	35.69	0.26	0.00–0.72
11	41.68	188	30.73	0.16	1 (bp)–5.07	10.95	0.06*	2 (bp)–0.78	114.51	0.61	0.00–5.73
Total	473.46	2084	349.19	0.17	1 (bp)–8.81	124.27	0.06	2 (bp)–4.65	1423.56	0.68	0.00–20.24

## Results

### Whole-genome re-sequencing and SNP identification

Whole genome re-sequencing of the parental lines ‘SZ41’ and ‘S1419’ yielded a total of 123 387 552 and 127 564 226 clean reads, respectively, corresponding to ~18.42 Gb and ~19.04 Gb of data, respectively. Of the clean reads from ‘SZ41’ and ‘S1419’, 99.51% and 98.52%, respectively, were successfully mapped to the cowpea reference genome, with 93.13% and 93.14% coverage, respectively (Table S1, see online supplementary material). The sequencing depths of ‘SZ41’ and ‘S1419’ were 34.92-fold and 35.43-fold, respectively (Table S1, see online supplementary material). Compared with the reference genome, a total of 1 472 234 and 1 323 311 SNPs were identified in ‘SZ41’ and ‘S1419’, respectively; furthermore, there were 362 715 homozygous SNPs detected between ‘SZ41’ and ‘S1419’, demonstrating a great deal of genetic difference between the two parents of the RIL population.

Whole-genome sequencing was also conducted for each of the 211 RILs, generating a total of 4 823 995 794 clean reads (~719.84 Gb). There were an average of 22 862 539 reads (~3.41 Gb) per line, equivalent to ~6.45-fold sequencing depth of the cowpea genome (Table S1, see online supplementary material). Compared with the reference genome, there were an average of 648 063 SNPs per line. After filtering, there were 514 227 statistically significant SNPs. Chromosomal distribution analysis revealed a minimum of 7867 SNPs per chromosome (Chr.06) and a maximum of 81 052 (Chr.05) (Table S2, see online supplementary material).

### Genetic map creation

**Figure 1:**
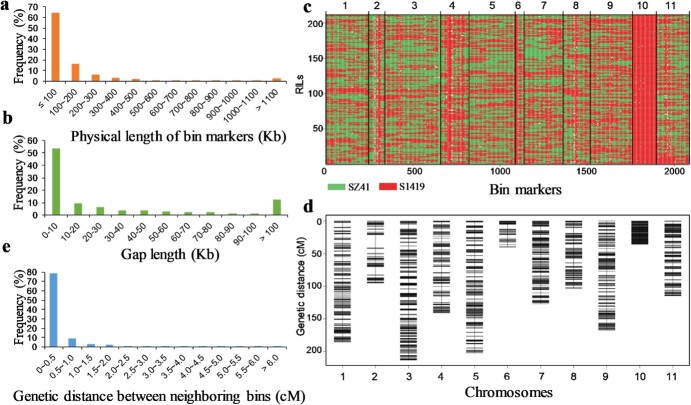
The RIL bin map. **a** Distribution of marker physical lengths in the RIL population. **b** Distribution of marker gap lengths in the RIL population. **c** Genotype data for 211 RILs. **d** Genetic map composed of bin markers on 11 chromosomes of the RIL population. **e** Distribution of the genetic distances between neighboring bins in the RIL population.

A total of 514 227 SNPs were used to generate a bin map. These corresponded to 2084 bin markers in the 11 chromosomes using a sliding window of 100 Kb (Table 1; Table S3, see online supplementary material). Map quality was assessed based on marker length and the gap sizes between neighboring bins. The physical bin marker length totaled 349.19 Mb; each marker ranged in size from 1 bp to 8.81 Mb, with an average of 167.56 Kb (Fig. 1a, Table 1). A total of 64.78% of the bins (1350) were shorter than 100 Kb, and 2.45% (51) were longer than 1.1 Mb. The largest bin (bin1) was located on Chr.06 (Fig. 1b; Table S3, see online supplementary material). The total gap length was 124.27 Mb, ranging from 2 bp to 4.65 Mb and averaging 59.66 Kb. The total gap length represented 26.25% of the total genome length (Table 1). A total of 53.55% of the gaps (1110) were shorter than 10 Kb, and 12.49% of the gaps (259) were longer than 100 Kb. The largest gap was located on Chr.04, between bin68 and bin69 (Fig. 1c; Table S3, see online supplementary material).

A bin marker matrix was next constructed for the RILs. This showed that 47.20% of all genotypes were inherited from the maternal parent, ‘SZ41’; 51.55% were inherited from the paternal parent, ‘S1419’; and 1.25% were heterozygous. The segregation ratios of each bin marker (‘SZ41’: ‘S1419’) ranged from 0 to 25.38, indicating the presence of significantly distorted segregation regions. A total of 119 bins showed extreme segregation distortion, with segregation ratios <0.01. These were primarily distributed on Chr.02, Chr.04, Chr.06, and Chr.10, with the largest number (94) on Chr.10 (Fig. S1, see online supplementary material).

A high-resolution genetic map was generated by mapping the 2084 bin markers onto 11 chromosomes, over which they spanned a total genetic distance of 1423.56 cM (Fig. 1d, Table 1). The average distance between neighboring bins was ~0.68 cM, ranging from 0.0 to 20.24 cM. A total of 78.69% of the regions (1640) were < 0.5 cM, and 0.91% (19) were >6.0 cM. There were only three regions greater than 10.0 cM. These were located on Chr.02 (bin36 and bin71) and Chr.04 (bin133) and were 20.24 cM, 12.41 cM, and 10.19 cM in length, respectively (Fig. 1e; Table S3, see online supplementary material). Most of the bin markers on all 11 chromosomes showed high collinearity between the physical and genetic maps (Fig. S2, see online supplementary material), demonstrating generally high quality and reliability.

### Phenotypic variation among RILs

There were notable differences in the measured traits between the two RIL parents (Table 2; Fig. S3, see online supplementary material). ‘SZ41’ had a purplish-red pod, whereas ‘S1419’ pods were white (Fig. S3, see online supplementary material). Transgressive segregation was observed at both extremes for all measured traits in each environment, indicating that positive alleles were present in both parental lines. Eight agronomic traits were assessed in the RIL population and the two parents in two different years: ANT, VE, TSP, PL, HSW, SL, SW, and SCC. Except for SCC, all of the measured traits displayed patterns of continuous variation consistent with quantitative traits (Fig. S4, see online supplementary material).

**Table 2:** Descriptive statistics of the seven analysed traits in the RIL population

Trait	Env.	Parent		RILs			
		P_1_	P_2_	Mean	Range	*GCV* (%)	*h^2^*(%)
ANT (ug/g)	2020	91.55	8.78	68.88	5.49–278.83	104.71	98.63
	2021	114.99	10.78	67.89	4.44–375.39	107.86	
	Combined	103.27	9.78	68.39	4.97–327.11	104.82	
VE (μg/g)	2020	89.39	63.86	89.96	18.75–197.79	34.88	81.18
	2021	94.08	67.45	65.59	11.65–124.37	40.23	
	Combined	91.74	65.65	77.77	15.20–161.08	31.24	
TSP (mg/g)	2020	1.84	0.39	0.77	0.15–1.82	39.05	82.94
	2021	1.13	0.31	0.83	0.25–1.48	30.25	
	Combined	1.49	0.35	0.80	0.29–1.60	30.54	
PL (cm)	2020	61.52	44.21	56.11	39.22–96.13	10.17	91.40
	2021	64.14	46.85	40.40	40.33–79.67	14.25	
	Combined	62.83	45.53	48.30	39.78–82.73	9.75	
HSW (g)	2020	10.99	16.35	16.30	11.01–23.22	12.90	51.80
	2021	16.57	18.58	18.19	12.36–37.96	16.73	
	Combined	13.78	17.47	17.25	12.59–26.72	12.46	
SL (mm)	2020	9.84	9.98	11.19	8.59–13.21	5.86	50.17
	2021	9.19	8.37	9.61	7.88–11.42	7.24	
	Combined	9.52	9.17	10.41	8.23–11.85	5.33	
SW (mm)	2020	5.77	5.74	6.26	5.20–7.02	5.45	52.43
	2021	5.15	5.08	5.21	4.28–6.47	7.82	
	Combined	5.46	5.41	5.74	4.75–6.70	5.46	

For ANT, the coefficient of variation (*CV*) was as high as 104.82%. The *CV* values for VE, TSP, PL, HSW, SL, and SW were 31.24%, 30.54%, 9.75%, 12.46%, 5.33%, and 5.46%, respectively, in the combined environment (Table 2). All traits possessed remarkably high broad-sense heritability (*h^2^*) in the combined environment, ranging from 50.17% (SL) to 98.62% (ANT). This indicated that all target traits had high selection efficiency.

### High-density-marker QTL mapping

**Figure 2:**
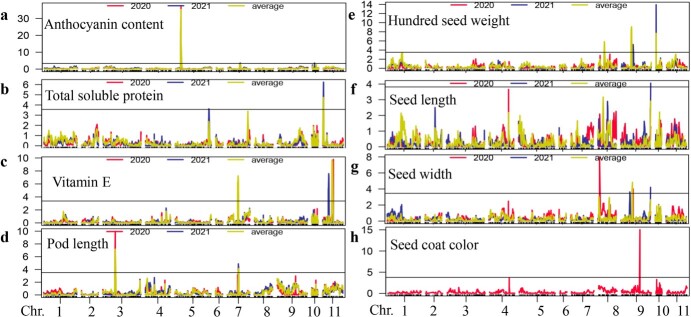
Identification of QTLs related to target traits via linkage mapping in yardlong bean. QTLs for (**a**) pod anthocyanin content; (**b**) vitamin E; (**c**) total soluble protein; (**d**) pod length; (**e**) hundred seed weight; (**f**) seed length; (**g**) seed width; and (**h**) seed coat color. The solid black line indicates the log-of-odds (LOD) significance threshold for QTLs as determined with permutation tests (1000×; *P* < 0.05).

Linkage mapping with the 2084 bin markers yielded a total of 20 QTLs for the eight analysed traits (Fig. 2, Table 2). Three QTLs on Chr.05, Chr.07, and Chr.10 were related to ANT, with LOD scores ranging from 3.61–37.64 (Table 2). The phenotypic variation was between 7.58% and 56.03%. The three QTLs showed positive additive effects, suggesting that the three alleles possessed by ‘SZ41’ increased ANT. The QTL *qANT5* could be detected in every individual and in the combined environment; it explained 56.03% of the phenotypic variation (Fig. 2a), indicating that it was a major QTL for anthocyanin accumulation.

Two QTLs on Chr.05 and Chr.11 were identified for TSP (Fig. 2b) and had LOD values of 3.63 and 6.24, respectively (Table 3). These explained 7.6% and 12.73% of the phenotypic variation, respectively. They had negative additive effects, implying that these two alleles from ‘S1419’ functioned to improve TSP values. One locus, *qTSP11*, was identified in the 2020, 2021, and combined environments simultaneously. Three QTLs for VE (Fig. 2c) were detected on Chr.07 and Chr.11. The LOD values ranged from 5.84 to 9.77 (Table 3) and they explained 11.96–19.20% of the phenotypic variation. All three of these QTLs had positive additive effects, demonstrating that ‘SZ41’ contributed three positive alleles that increased VE content. *qVE7* was identified in every environment, both individual and combined.

**Table 3:** Key QTLs mapped for eight agronomic traits in the RIL population

Trait	LOD cutoff	QTL	Chr.	Leading Marker	Interval (bp)	Position (cM)	Env.	LOD	*R^2^* (%)
ANT	3.31	*qANT5*	5	bin45	3 039 317–3 208 796	29.85	2020, 2021, combined	37.64	56.03
		*qANT7*	7	bin132	21 732 367–21 927 304	68.98	2020	3.61	7.58
		*qANT10*	10	bin45	10 600 962–10 658 655	12.16	2021	3.61	7.59
TSP	3.56	*qTSP5*	5	Bin252	44 171 737–44 175 927	188.46	2021	3.63	7.60
		*qTSP11*	11	Bin5	1 674 363–1 677 173	4.61	2020, 2021, combined	6.24	12.73
VE	3.37	*qVE7*	7	Bin119	17 493 571–17 493 571	59.61	2020, 2021, combined	5.84	11.96
		*qVE11-1*	11	Bin74	26 332 230–26 531 778	33.73	2021, combined	7.58	15.26
		*qVE11-2*	11	Bin108	31 973 563–32 031 190	59.80	2020	9.77	19.20
PL	3.51	*qPL3*	3	Bin96	16 734 572–16 793 335	70.19	2020, 2021, combined	7.19	14.52
		*qPL7*	7	Bin123	17 898 486–18 040 234	63.17	2021, combined	4.88	10.10
HSW	3.51	*qHSW8*	8	Bin53	7 206 431–7 376 447	33.83	combined	5.85	11.98
		*qHSW9-1*	9	Bin82	8 360 268–8 843 117	57.27	2020, combined	9.11	18.02
		*qHSW9-2*	9	Bin105	14 666 633–15 429 694	65.88	2021	5.20	10.73
		*qHSW10*	10	Bin11	4 767 804–4 907 598	2.37	2021, combined	13.93	26.22
SL	3.82	*qSL9*	9	Bin244	43 205 001–43 933 251	167.11	2021	4.06	8.48
SW	3.46	*qSW8*	8	Bin24	1 483 708–1 492 887	10.22	2020	7.94	15.92
		*qSW9-1*	9	Bin73	6 657 233–6 741 444	51.10	2021	3.63	7.63
		*qSW9-2*	9	Bin105	14 666 633–15 429 694	65.88	combined	4.84	10.00
		*qSW9-3*	9	Bin244	43 205 001–43 933 251	167.11	2021	4.21	8.79
SCC	3.78	*qSCC9*	9	Bin152	31 106 322–31 310 082	103.16	2020, 2021, combined	15.42	28.58

Chr.: chromosome; Env.: environment; LOD, log-of-odds; QTL, quantitative trait loci.

The threshold LOD score for major QTLs was determined with a 1000× permutation test (*P* < 0.05).

Linkage mapping was also used to explore loci associated with PL, HSW, SL, SW, and SCC, for which we detected two, four, one, four, and one QTLs, respectively (Fig. 2d–h and Table 3). Two QTLs, located on Chr.03 and Chr.07, were associated with PL; *qPL3* was detected in both environments and in the combined environment, explaining 14.52% of the phenotypic variation (Table 3). These results indicated that *qPL3* was a major QTL for PL in this RIL population. Notably, *qSCC9* was an important QTL for seed coat color; it had a LOD of 15.42 and explained 28.58% of the phenotypic variation.

### Identification of a putative causal gene for ANT

**Figure 3:**
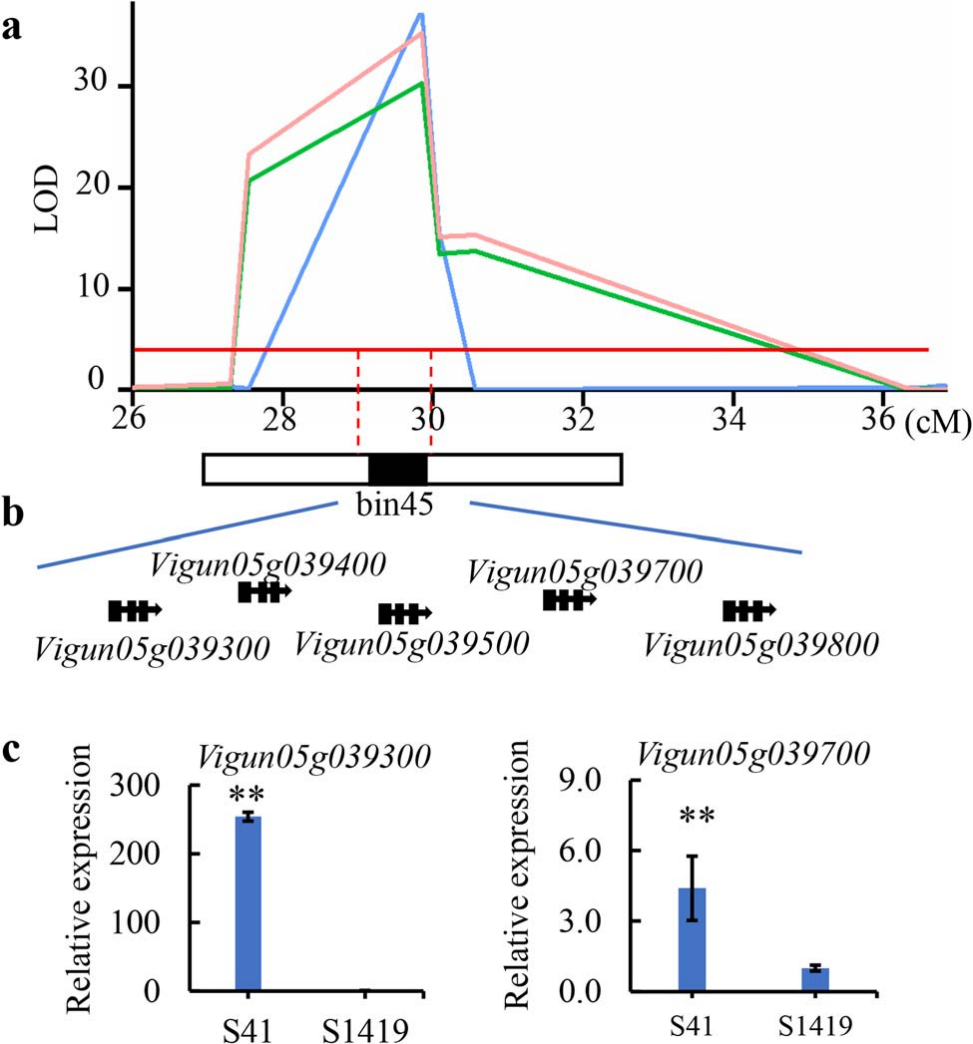
Identification of a putative causal gene for *qANT5*. **a** A major QTL (bin45) related to anthocyanin content was identified on Chr.05 with linkage mapping. **b** Five genes (*Vigun05g039300*, *Vigun05g039400*, *Vigun05g039500*, *Vigun05g039700*, and *Vigun05g039800*) predicted to encode MYB113 transcription factors were located in bin45. **c** Relative expression of *Vigun05g039300* and *Vigun05g039700* in immature ‘SZ41’ and ‘S1419’ pods. ^**^P < 0.01.

*qANT5* was associated with ANT in both seasons and in the combined environment. The representative marker bin45 spanned a genomic region of 169 Kb (from 3039,317 bp to 3 208 796 bp on Chr.05) (Fig. 3a) and explained 56.03% of the phenotypic variation. This indicated that *qANT5* was a major QTL for anthocyanin content in this RIL population. Subsequent analysis showed that *qANT5* overlapped with a previously reported locus related to pod color and pod tip color in cowpea [17, 18]. The annotated cowpea genome (v1.2) showed 20 predicted genes in this region, five of which encoded MYB113 transcription factors (TFs): *Vigun05g039300*, *Vigun05g039400*, *Vigun05g039500*, *Vigun05g039700*, and *Vigun05g039800* (Fig. 3b). *MYB* genes are known to be regulators of anthocyanin biosynthesis [19–24]. The identified *MYB113* genes were therefore considered strong candidates for ANT concentrations. qRT-PCR was performed to investigate the expression patterns of these five genes in the immature pods of ‘SZ41’ and ‘S1419’ individuals. Three of the genes (*Vigun05g039400*, *Vigun05g039500*, and *Vigun05g039800*) were not expressed at detectable levels in the developing pods of ‘SZ41’ and ‘S1419’. However, *Vigun05g039700* was expressed ~4.5-fold higher in the purple pods of ‘SZ41’ than in the white pods of ‘S1419’, and *Vigun05g039300* was expressed ~254-fold higher in ‘SZ41’ than in ‘S1419’ (Fig. 3c).

Based on the significant differential expression of *Vigun05g039300* between ‘SZ41’ and ‘S1419’, we hypothesized that there may have been cultivar-specific differences in the *Vigun05g039300* promoter. We therefore sequenced the *Vigun05g039300* promoter region (defined as the sequence ~2 kb upstream of the start codon) from ‘SZ41’ and ‘S1419’ to explore possible sequence variations. This analysis revealed a 3-bp insertion and 32-bp, 5-bp, and 4-bp deletions at −1093 bp, −1412 bp, −1759 bp, and −1825 bp, respectively, in ‘SZ41’ compared to ‘S1419’. In addition, 18 SNPs were also detected in the *Vigun05g039300* promoter between ‘SZ41’ and ‘S1419’. The same insertion and deletions were detected in RILs with purple pods compared to those with white pods. Furthermore, RILs with purple pods showed higher expression of *Vigun05g039300* than RILs with white pods. Together, these results indicated that *Vigun05g039300* may have been the causal gene in the major anthocyanin-regulating QTL *qANT5*. We therefore designated the gene *VuANT1* and selected it for additional characterization experiments.

### *VuANT1* regulated anthocyanin biosynthesis

**Figure 4:**
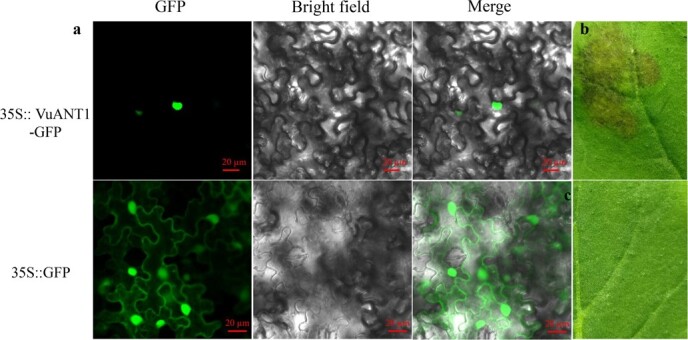
**a** Subcellular localization of *VuANT1 in Nicotiana tabacum.***b** At 4–5 d after infiltration with a 35S::VuANT1 construct, tobacco leaves changed color. **c** A blank control tobacco leaf infiltrated with 35S::GFP.

To characterize *VuANT1*, we first determined the subcellular localization of a VuAN1-GFP fusion protein with confocal microscopy. These experiments showed that it was exclusively localized to the nucleus (Fig. 4a). This was in contrast to the control 35S::GFP protein, which was localized to both the cytoplasm and the nucleus. To establish the role of *VuANT1* in anthocyanin biosynthesis, a *VuANT1* construct was transiently expressed in tobacco leaves. At 4 d after injection with the *VuANT1* construct, the leaves showed visible anthocyanin accumulation (Fig. 4b), whereas leaves injected with the empty 35S::GFP vector did not show any change in color (Fig. 4c).

**Figure 5:**
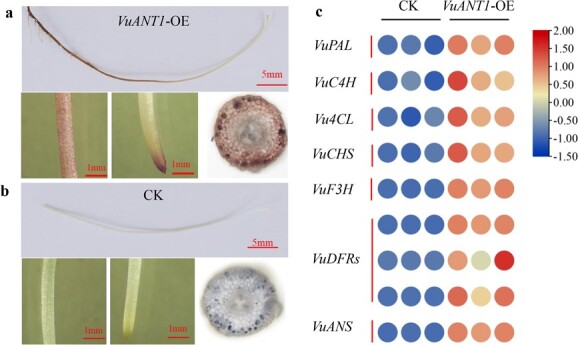
Functional validation of *VuANT1*. **a** Transient expression of 35S::VuANT1 induced purple hairy roots in yardlong bean (*VuANT1*-OE). **b** Negative control (CK). **c** Relative expression levels of anthocyanin synthesis-related genes in yardlong bean hairy roots (CK and *VuANT1*-OE). *VuPAL*: *Vigun01g160400*; *VuC4H:Vigun08g135200; Vu4CL: Vigun02g189900; VuCHS: Vigun01g080800; VuF3H: Vigun03g258800; VuDFRs: Vigun07g040600, Vigun08g013800,* and *Vigun08g013900; VuANS: Vigun02g171400.* Three independent lines of yardlong bean hairy roots were used in gene expression analysis.

Yardlong bean hairy roots were induced and used to further confirm the role of *VuANT1* in anthocyanin biosynthesis. Ectopic *VuAN1* expression (*VuANT1*-OE) in hairy roots induced anthocyanin accumulation and caused a dark purple color in the hairy roots (Fig. 5a), whereas roots transformed with the empty vector (CK) remained white (Fig. 5b). To determine how *VuANT1* regulated anthocyanin biosynthesis in cowpea, we conducted qRT-PCR to quantify expression levels of structural anthocyanin genes during anthocyanin synthesis in the hairy roots. Analysis of genes encoding phenylalanine aminotransferases (PALs), cinnamic acid-4-hydroxylases (C4Hs), flavanone 3-hydroxylases (F3Hs), dihydroflavonol-4-reductases (DFRs), and anthocyanidin synthases (ANSs) showed that these structural genes were expressed significantly higher levels in the purple (*VuANT1*-overexpressing) hairy roots than in the white (control) hairy roots (Fig. 5c). Together, these findings suggested a regulatory function of *VuANT1* in anthocyanin accumulation, in which it modulated expression of anthocyanin structural genes.

## Discussion

Yardlong bean is an important legume that is widely planted in China. The primary factors affecting the quality of this plant and its desirability to consumers are pod appearance and the levels of soluble proteins, fiber, vitamin E, and anthocyanins in the beans and pods. To engineer more productive cultivars with optimal traits, it is necessary to understand the genetic bases of key agricultural traits. Such studies require high-quality, high-density genetic maps. Indeed, in recent years, genetic maps have been constructed using a variety of sequencing technologies to explore loci responsible for target traits in cowpea. For example, five genetic maps have been constructed for cowpea from bi-parental RIL populations via whole-genome resequencing; each map includes 7964–16 578 SNPs distributed among 697–1083 genetic bin markers [25]. Li *et al.* [26] generated a high-density genetic map based on 9493 SNPs via specific-locus amplified fragment sequencing to identify loci associated with pod color. In another study, Pan *et al.* [27] used the restriction-site associated DNA (RAD) sequencing technique on a population including 170 *V. unguiculata* individuals, comprising two parents and their progeny. That study identified 17 996 reliable SNPs on 11 consensus linkage groups (LGs) and constructed a genetic map totaling 1194.25 cM [27].

Maps such as these have been used to identify the genetic bases of agronomically important traits such as seed weight, SL, SW, PL, PW, pod color, and pod fiber contents. In these studies, QTLs, SNPs, and candidate genes associated with the target traits have been detected with linkage analyses, genome-wide association studies, or transcriptomic analyses [17, 18, 28–30]. For example, major QTLs have been identified in cowpea for seed, pod, stem, and leaf size [29, 6], PT, PL, TSSC, pod dehiscence [11], and pod fiber content [30].

Here, we generated a high-quality genetic map containing 2084 bin markers (514 227 SNPs) from a RIL population designed to identify QTLs for yardlong bean pod color (Fig. S1, see online supplementary material). Using this map, we identified 20 QTLs for eight agronomically important traits using a linkage mapping approach. One of these traits was PL, which is particularly important because consumers in Asia often eat fresh yardlong bean pods. We identified the PL QTL *PL3* in both growth seasons and the combined environment, and it explained 18.02% of the phenotypic variation (Table 2). These results suggested that *PL3* was an important genomic region for pod development in yardlong bean. Other studies have identified QTLs or SNPs related to PL using linkage analyses or GWAS [29, 31, 32], but the previously identified regions did not overlap with *PL3*. This indicated that *PL3* is a novel PL QTL; the differences in identified loci may have been due to differences in the cultivars analysed. As measures of organ size, we also quantified and identified QTLs associated with HSW, SL, and SW in the RIL population. Interestingly, both the HSW QTL *qHSW9–2* and the SW QTL *qSW9–2* mapped to a single region in Chr.09, and both the SL QTL *qSL9* and the SW QTL *qSW9–3* mapped to a single region in Chr.09. These results demonstrated that some loci were associated with two or more traits simultaneously, which could explain pleiotropic effects and correlations between these traits.

As mentioned above, a key trait of yardlong bean that affects consumer preference is pod color. Anthocyanins impart coloration to the pods of cowpea plants and confer health benefits ([9]. We here identified a key QTL for anthocyanin accumulation in yardlong bean pods, *qAN5*. Other studies have also identified this as an important region for anthocyanin biosynthesis [17, 18, 26]. For example, the locus *pc* on Chr.05 has been shown to control cowpea pod color [18], and four QTLs responsible for seed coat and pod tip color in yardlong bean map to the same region on Chr.05 [17].

Like all plant specialized metabolites, biosynthesis of anthocyanin is controlled by structural and regulatory genes. MYB-domain TFs are known to regulate the last steps of anthocyanin biosynthesis [17, 21, 22, 26]. For example, a cluster of six MYB TFs is responsible for anthocyanin accumulation in carrot roots and petioles [33]. Analysis of the genomic region corresponding to *qAN5* revealed a cluster of five MYB TFs: *Vigun05g039300*, *Vigun05g039400*, *Vigun05g039500*, *Vigun05g039700*, and *Vigun05g039800*. Furthermore, genomic comparison of a purple-pod and a green-pod cowpea cultivar showed that the latter was missing a segment of ~40–42 kb. This deletion removed two MYB genes entirely (*Vigun05g039400* and *Vigun05g039500*) and truncated two others (*Vigun05g039300* and *Vigun05g039700*) [17].

To determine if any of the five MYBs in *qAN5* were responsible for regulating anthocyanin biosynthesis, we analysed the expression patterns of each. Three of the *MYB* genes (*Vigun05g039400*, *Vigun05g039500*, and *Vigun05g039800*) were too lowly expressed to be detected in the developing pods of ‘SZ41’ and ‘S1419’. Of the five genes, *Vigun05g039300* showed the most significant change in mRNA abundance between ‘SZ41’ and ‘S1419’; this gene was expressed at almost undetectable levels in the white-pod line ‘S1419’ (Fig. 3c) and was expressed nearly 254-fold higher in ‘SZ41’. This finding was in contrast to expression levels of *Vigun05g039700*, which was highly expressed not only in the purple-pod cultivar ‘SZ41’ but also in the white-pod cultivar ‘S1419’. There was only a difference of ~4.5-fold in *Vigun05g039700* expression between the pods of the two cultivars. In general, a candidate gene that shows highly differential expression between accessions that differ with respect to the target trait is likely to be a causal gene. *VuANT1* was therefore considered a causal gene for the QTL *qAN5*, which regulated anthocyanin accumulation. This hypothesis was functionally confirmed with *VuANT1* overexpression in tobacco leaves and cowpea hairy roots, in which it increased anthocyanin expression (Figs 4 and 5). *Vigun05g039700* also showed differential expression between ‘SZ41’ and ‘S1419’, and may therefore participate in anthocyanin biosynthesis. Actually, a transient expression of *Vigun05g039700* was taken in tobacco leaf, and resulted in visible anthocyanin accumulation around the injection site after 4 days (Fig. S1a, see online supplementary material), whereas leaves injected with the empty 35S::GFP vector did not show any change in color (Fig. S1b, see online supplementary material). This result suggested that *Vigun05g039700* may have a similar function as the *Vigun05g039300*, both of which regulate the anthocyanin accumulation in yardlong bean. However, more experimental evidence will be required to confirm this hypothesis in the future.

Within *qAN5*, *VuANT1* was present in a cluster of *MYB113* genes. These genes are relatively abundant and closely evolutionarily related in the cowpea genome. It is therefore unknown whether *VuANT1* acts alone or whether a functionally redundant duplicate gene may also regulate anthocyanin accumulation in yardlong bean. Genetic redundancy is typically verified by analysing the phenotypes of single mutants, which produce mildly abnormal phenotypes, and the corresponding higher-order mutants, which exhibit more strongly abnormal phenotypes. However, genetic transformation in yardlong bean remains difficult, complicating such analyses. Future studies should focus on the use of technologies such as CRISPR/Cas9 to generate sets of single and higher-order mutants, allowing deeper exploration of the regulatory mechanisms associated with genes that control anthocyanin production and accumulation.

In summary, we here generated a high-density linkage map for cowpea and detected QTLs related to PL, HSW, SL, SW, and pod color. These loci will aid in development of molecular markers for future breeding efforts using marker-assisted selection (MAS). We also characterized the genes present in a QTL for pod color and positively identified a gene that promoted anthocyanin biosynthesis in cowpea and tobacco. This study not only deepens our understanding of the molecular mechanisms controlling anthocyanin biosynthesis in plants but also provides a valuable resource for future studies seeking to map QTLs, locate genes, or perform comparative genomic analyses for agriculturally important traits in this economically important species.

## Materials and methods

The subject of this study was an F_7_-derived RIL population from a cross between the purple-podded *V. unguiculata* cultivar ‘Suzi 41’ (‘SZ41’) (Fig. S1a, see online supplementary material) and the white-podded cultivar ‘Sujiang 1419’ (‘S1419’) white (Fig. S1b, see online supplementary material). This population comprised 211 lines (Fig. S1c, see online supplementary material). All lines were planted in a completely randomized block design at the Experiment Station for Animal Science (32.5° N 118.6° E) in Nanjing, China in 2020 and 2021. Each ridge comprised two rows with one bunch (containing two plants) every 1 m. Field management was consistent with standard protocols. Biotic and abiotic stressors were minimized with regular fertilization and herbicide application. Pods were collected and snap-frozen in liquid nitrogen, then stored at 4°C prior to further analyses.

### Single nucleotide polymorphism (SNP) identification

Fresh leaves were collected from the 211 RILs and parental lines. Genomic DNA was extracted as previously described [34]. Single-indexed Illumina libraries were constructed using a TruSeq Nano DNA LT Sample Prep Kit (Illumina, San Diego, CA, USA) following the manufacturer’s instructions. Paired-end libraries were sequenced by Shanghai Biozeron Biotechnology Co., Ltd (Shanghai, China) on the Illumina HiSeq PE platform (2 × 151 bp reads). After the raw reads were determined to be of sufficient quality for subsequent analyses, they were aligned to the cowpea reference genome (https://phytozome.jgi.doe.gov) using BWA software [35]. The sequencing depth and reference genome coverage were calculated from the alignment, then SNP calling was performed with SAM tools [36]. The resulting SNPs were filtered using custom scripts to retain only candidate SNPs at *P* > 0.05 that were homozygous in both parents and in the associated RIL.

### Bin map construction

A bin map was generated from the final filtered SNP dataset using the maximum parsimonious inference of recombination (MPR) method [37]. Consistent with MPR, SNP sites with the same genotypes were identified as a block. A recombination event was defined as a transition of two genotypes. A chromosome interval with the same genotype across each population was recorded as a bin. Data were processed for bin map construction as follows: (i) SNP re-identification in permutations generated by resampling the SNP windows; (ii) SNP inference with a Bayesian method; (iii) RIL genotype determination at each SNP locus with a hidden Markov model; (iv) collection of consecutive SNPs with the same genotype as one parent into one block using the sliding window method; and (v) marking windows with <100 kb blocks or <20 SNPs as missing data. The genetic map was constructed using the ‘qtl’ package (http://rqtl.org/) in R.

### Biochemical analyses

Anthocyanin content was determined as described by Wang [38]. Briefly, yardlong bean pods were weighed, homogenized in 10 mL of 1% HCl in methanol (v/v) per g of fresh weight (FW), then incubated overnight at 4°C in the dark. A 3:2:2 methanol:water:chloroform solution was prepared, then samples were mixed well with the solution and centrifuged at 3000 × g for 10 min. The absorbance values of the methanol–water phase were measured at 530 nm and 657 nm on a T6 New Century UV–Vis spectrophotometer. Cyanidin-3-glucoside chloride was used as an internal reference. Anthocyanin concentrations were then calculated as follows [39]:

(1.1a) \begin{align*} \textrm{Anthocyanin}\ (\mu \textrm{g}/\textrm{g}\ \textrm{FW})= \textrm{A}_{530}-0.25\ *\ \textrm{A}_{657} \end{align*}

Vitamin E content was measured using a Vitamin E kit obtained from NanJing JianCheng Bioengineering Institute (Jiangsu, China) and expressed in μg/g FW. The total soluble protein content was determined spectrophotometrically using Coomassie blue G-250 as described by Bradford [40] and expressed in mg/g FW.

### Phenotyping

All phenotypic data were expressed as the mean ± standard deviation from three technical replicates. Statistics and correlation analyses were conducted in SAS (version 9.0) and SPSS (version 17.0), respectively, using the mean value of each trait over three years.

### Linkage analysis

QTL mapping was implemented using the composite interval mapping (CIM) method [41, 42] in the R package ‘qtl’ (http://rqtl.org/). The permutation time was set to 1000 and the significance threshold was 0.05. We analysed the identified QTLs to determine the chromosome, marker, position, confidence interval, LOD value, and proportion of phenotypic variance explained (*r*^2^). Each identified QTL was named following the method of Cui et al. [43]: names began with *q*, followed by an abbreviation of the trait name, the chromosome number, and the number of the QTL in order on the chromosome. The maximum LOD score of the interval was considered the QTL location. The region from LOD – 1 to LOD + 1 was defined as the confidence interval.

### Real-time PCR

RNA was isolated using an RNA simple Total RNA Kit (Tsingke, Beijing, China). First-strand cDNA was reverse-transcribed with a Goldenstar™ RT6 cDNA synthesis kit (Tiangen, Beijing, China). Real-time PCR reactions were carried out in SYBR Green Real-time Master Mix (Toyobo, Shanghai, China) on an ABI 7500 system (Applied Biosystems, Carlsbad, CA, USA) (for primer sequences, see Table S4, see online supplementary material).

### Subcellular localization

The full-length *VuANT1* sequence without the stop codon was cloned from ‘SZ41’ genomic DNA and inserted into the pCAMBIA1305-GFP vector under the control of the CaMV promoter. The resulting 35S::*VuANT1*-GFP construct was transformed into *Agrobacterium tumefaciens* strain EHA105, then infiltrated into four-week-old tobacco (*Nicotiana benthamiana* L.) leaves. The empty 35S::GFP vector was used as a blank control. GFP fluorescence was detected with the Zeiss LSM800 confocal microscope (Zeiss, Oberkochen, Germany).

### Hairy-root transformation

*Agrobacterium rhizogenes* strain K599 was transformed with 35S::*VuANT1*-GFP, then used to transform 1-week-old hairy roots of ‘SZ41’ seedlings as previously described [44]. The transgenic seedlings were transferred to a germination chamber and grown under a 12/12 h light/dark cycle (28/25°C with high humidity). After 2–3 weeks, when the hairy roots were ~2–10 cm closer to the infection site, the primary root was removed. The seedling hairy roots were then grown in ½ Hoagland nutrient solution for 2 weeks.

## Supplementary Material

Web_Material_uhad247

## Data Availability

The data supporting the findings of this work are available within the paper and its supplementary information files. The datasets generated and analysed during this study are available from the corresponding author upon request.
