# Supplementary material for: Construction of a high-density genetic map for yardlong bean and identification of ANT1 as a regulator of anthocyanin biosynthesis
Source: Hortic Res. 2023 Nov 27;11(1):uhad247. doi: 10.1093/hr/uhad247 (PMC10809905; doi:10.1093/hr/uhad247)
Supplement: Web_Material_uhad247 [file web_material_uhad247.zip › Supplementary materials.docx]

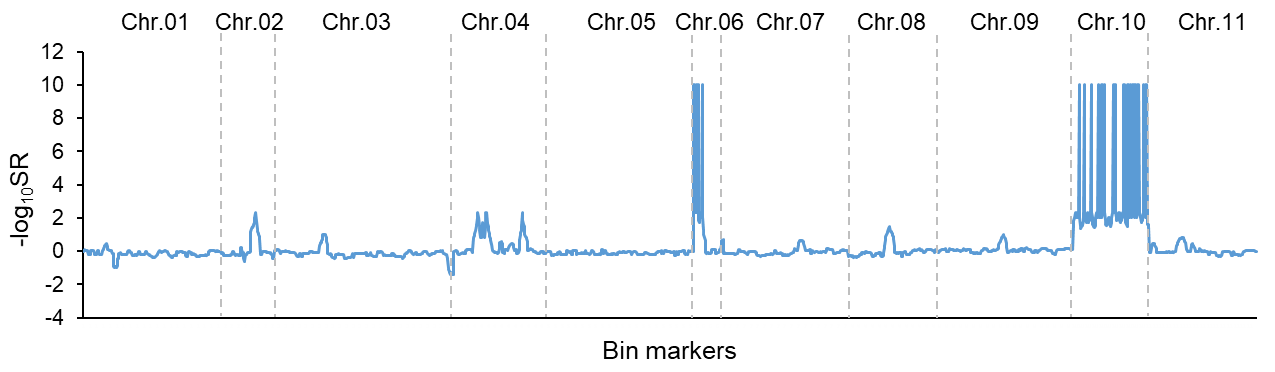


**Fig. S1** Negative log_10_ values of segregation ratios in the RIL population

**
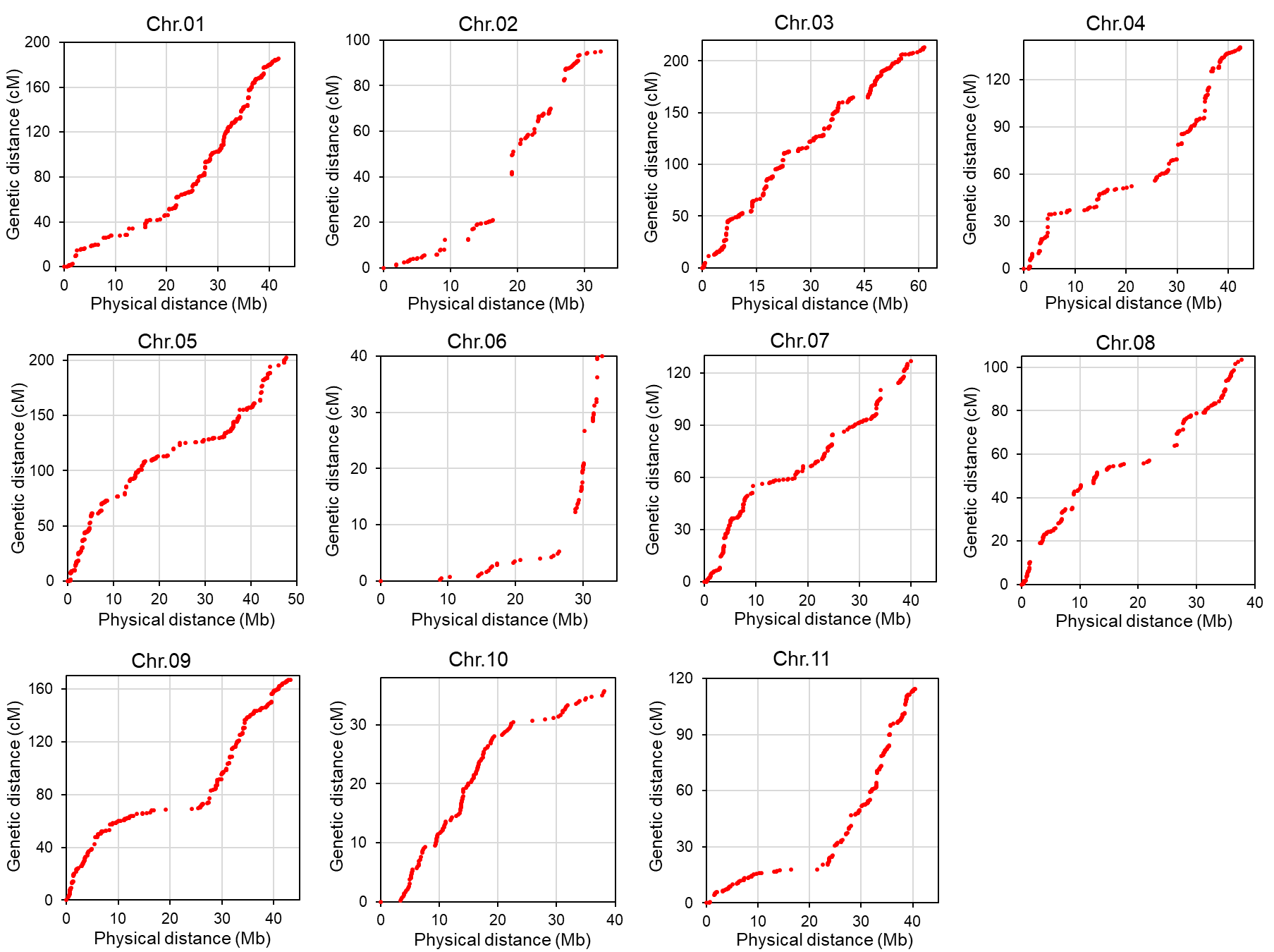
Fig. S2** Collinear analysis of the consensus between genetic and physical maps of the RIL population

The X axis indicates the physical position of each bin marker, the Y axis indicates the genetic position of each bin marker.


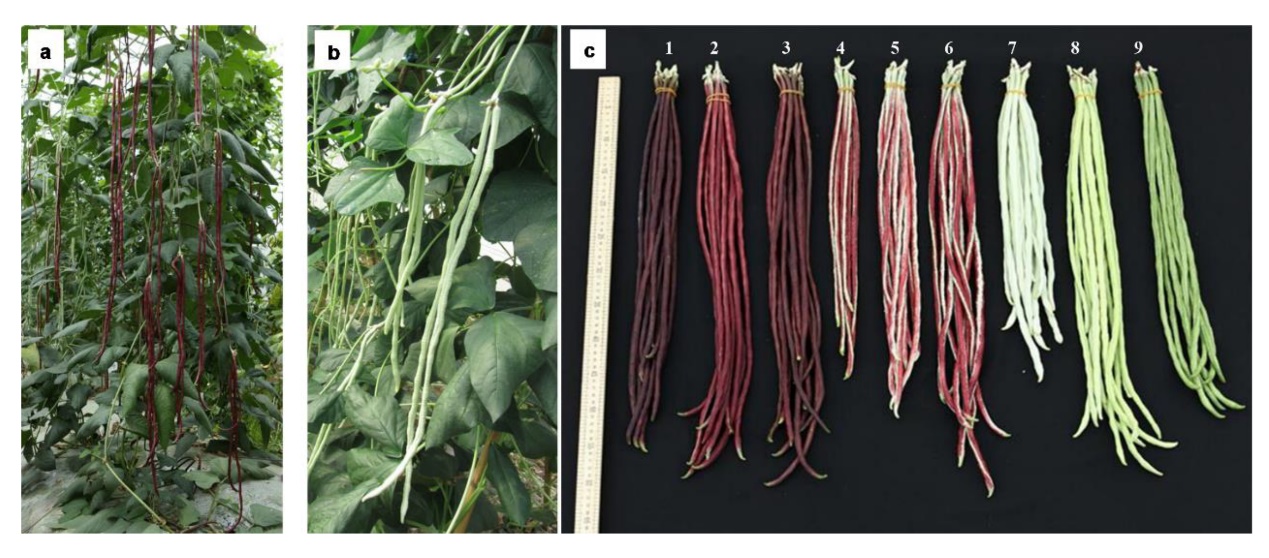


**Fig. S3** Phenotypic differences between the two parents and the RIL populations. (a) P_1_ Suzi41; (b) P_2_ Sujiang1419; (c) Some representative individuals of the RILs.


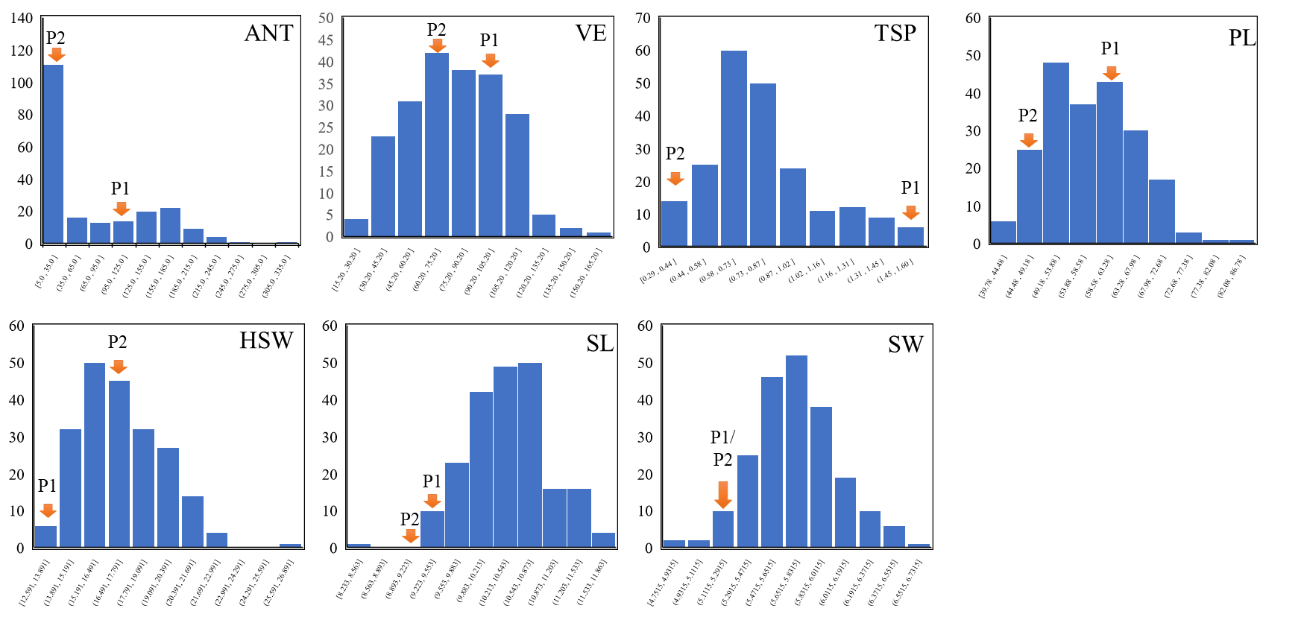


**Fig. S4** Frequency distributions of the seven traits among two environments in the RIL population


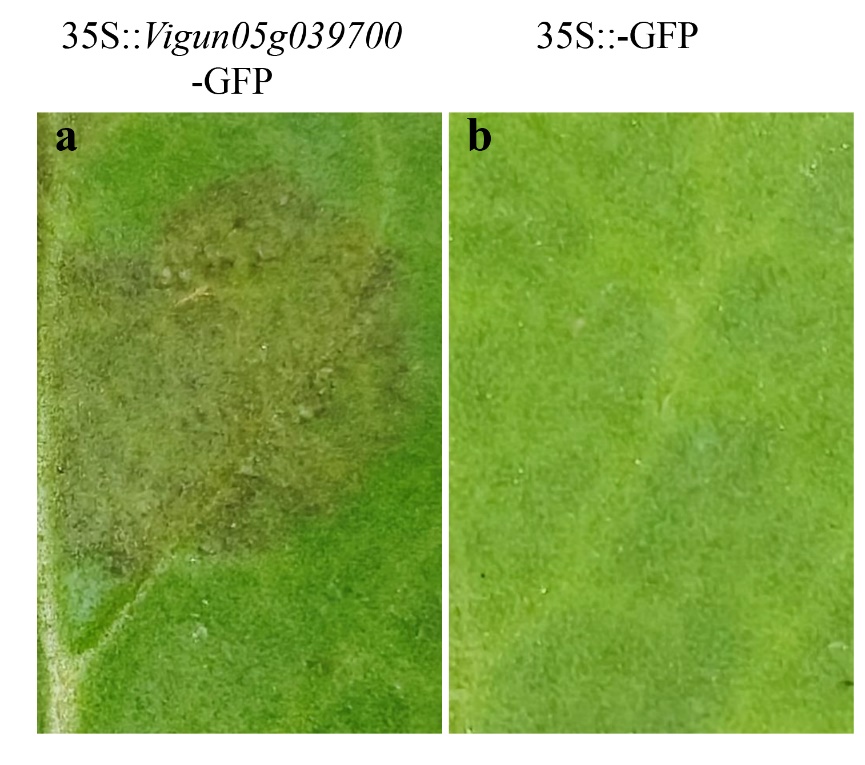


**Fig. S5** A transient expression of *Vigun05g039700* in tobacco leaf. (**a**) At 4 days after infiltration with a 35S:: *Vigun05g039700-*GFP construct, tobacco leaves changed color. (**b**) A blank control tobacco leaf infiltrated with 35S::GFP.
